# Dubuque Meeting of the American Association for the Advancement of Science

**Published:** 1872-07-01

**Authors:** H. T. Woodman, S. Calvin

**Affiliations:** Chairman; Dubuque, Iowa; Secretary; Dubuque, Iowa


### DUBUQUE MEETING OF THE AMERICAN ASSOCIATION FOR THE ADVANCEMENT OF SCIENCE.

### CIRCULAR OF THE LOCAL COMMITTEE.

The objects of the American Association for the Advancement of Science are, "by periodical and migratory meetings, to promote intercourse between those who are cultivating science in different parts of North America; to give a stronger and more general impulse, and a more systematic direction to scientific research in our country, and to procure for the labors of scientific men increased facilities and a wider usefulness."

The following extracts from the Constitution and Resolutions of the Association relate to membership:

*Rule* 1. Any person may become a member of the Association upon recommendation in writing by two members, nomination by the Standing Committee, and election by a majority of the members present.

*Resolution* 9. Associate members may be admitted for one, two, or three years, as they shall choose at the time of admission; to be elected in the same way as permanent members, and to pay the same dues. They shall have all the social and scientific privileges of members, without taking part in the business.

The Twenty-first Annual Meeting of the Association will be held at Dubuque, Iowa, commencing Wednesday, August 21, 1872, at 10 o'clock A.M.

On the evening of Wednesday, August 21, a reception will be extended to the Association by the Hon. Wm. W. Allison, U. S. Senator elect, and Chairman of the Committee of Reception. Response from the Association, after which Prof. Asa Gray, retiring President of the Association, will deliver his address and give up the chair to his successor.

From the success that has already attended the efforts of the Special Committees, and the expressed determination of the citizens to extend a liberal hospitality to the members, we can confidently promise that all can be entertained at private residences, *free of charge*, during the session.

The Local Committee, therefore, earnestly request those intending to be present to notify the Local Secretary by letter as soon as possible.

Members and those intending to become members will report immediately upon their arrival at the Reception Room of the Local Committee and register their names, when they will be conducted to the places to which they have been assigned.

Notice of the location of the Reception Room of the Local Committee will be posted at the railroad depots, steamboat landings, and in the street cars and omnibuses of the city.

Negotiations with the railroads have now progressed so far as to make it almost certain that we will be able to give return passes over all the principal lines.

By order of the Local Committee,

H. T. WOODMAN, *Chairman.*
S. CALVIN, *Secretary.*

DUBUQUE, IOWA, July 4, 1872.

